# Supplementary material for: Polar domain walls trigger magnetoelectric coupling
Source: Sci Rep. 2015 Sep 21;5:13784. doi: 10.1038/srep13784 (PMC4585675; doi:10.1038/srep13784)
Supplement: Supplementary Information [file srep13784-s1.pdf]

Supplementary Information

Polar domain walls trigger magnetoelectric coupling

Josep Fontcuberta[a], *Vassil Skumryev*[a,b,c], *Vladimir Laukhin*[a,b], *Xavier Granados*[a]
and
Ekhard K. H. Salje[d]

[a] Institut de Ciència de Materials de Barcelona (ICMAB-CSIC),
Campus UAB, 08193, Bellaterra, Catalonia, Spain.

[b] Institució Catalana Recerca & Estudis Avançats, 08010 Barcelona, Catalonia, Spain.

[c] Univ. Autònoma de Barcelona, Dept. Física, 08193 Bellaterra, Catalonia, Spain,

[d] Department of Earth Sciences, University of Cambridge, Downing Street, Cambridge
CB3 2EQ UK

Supplementary Information 1

Epitaxial strain on $La_{1/2}Sr_{1/2}MnO_3$ films on (001)STO and (001)LAO.

In Figure S1, reciprocal space maps of LSMO films of 20 nm, grown on STO(001) (a) and LAO(001) (b) substrates, recorded around the corresponding (113) reflections of the substrates, are shown.

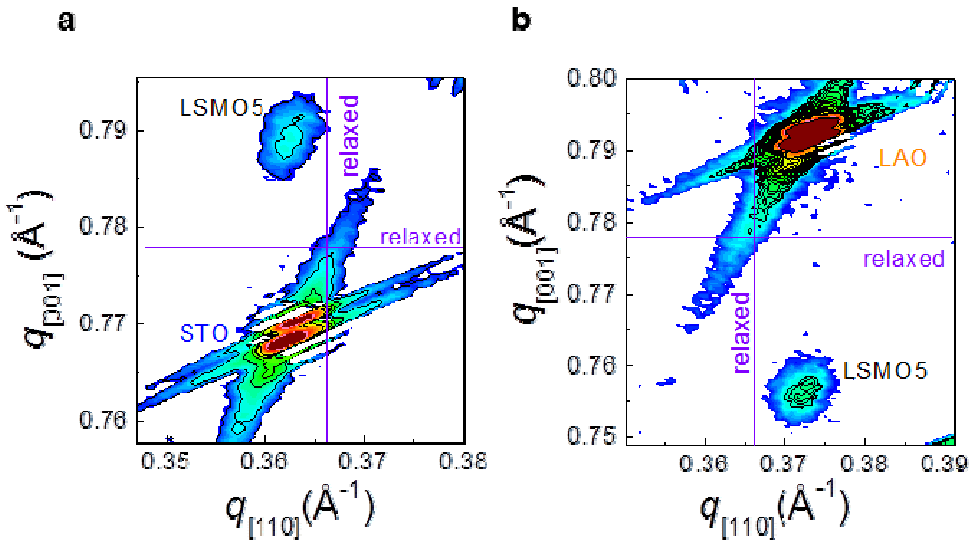

Figure S1. Reciprocal space maps of LSMO films. Around the reflection (113) of (a) STO and (b) LAO. The tensile and compressive strain state of the LSMO on (001) STO and LSMO on (001) LAO films are evidenced by the shift towards smaller (larger) *q*-values of the in-plane direction of reflections of LSMO when compared to the bulk value (vertical line).

Supplementary Information 2

Temperature dependence of the magnetization.

In Figure S2 a) we show the temperature dependence of the magnetization (M) of the LSMO(34 nm)//(001)STO film, measured using H-ZFC and H-FC protocols as indicated (H_{meas} = 100 Oe). It can be observed that the Curie temperature is of about 280 K. The characteristic changes of magnetization at the T_{CT} of STO can be appreciated in both measurements. Measurements have also been performed at larger magnetic field (500 Oe and 1000 Oe). In all cases the kink of the M(T) data at T_{CT} can be well appreciated although, as expected, the difference between the H-ZFC and H-FC curves gradually vanishes when increasing the measuring field. In Figure S2 b) we show the corresponding measurement on the LSMO(20 nm)/LAO sample. No visible changes can be appreciated around 105 K. In agreement with earlier reports, the magnetization of LSMO films on LAO is smaller and films are magnetically harder.

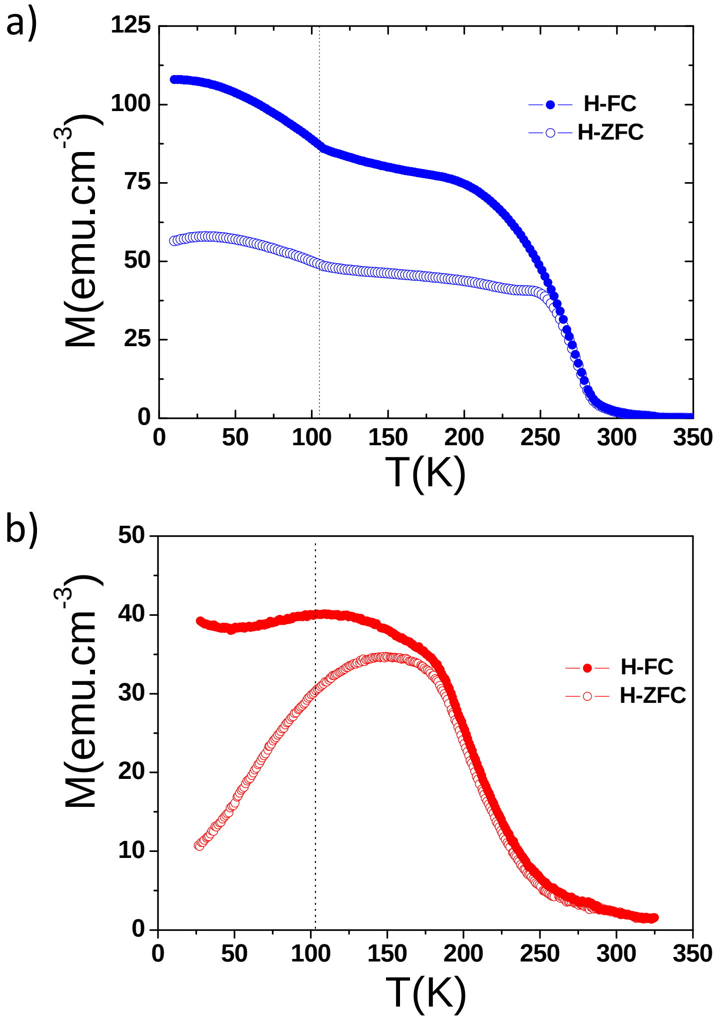

Figure S2. Temperature dependence of the magnetization of a) LSMO(34 nm)/(001)STO and b) LSMO(20 nm)/LAO film. Measurements performed under H_{meas} = 100 Oe, after cooling the sample either under magnetic field (H-FC) (solid symbols) or in nominally zero magnetic field (H-ZFC) (open symbols).

Supplementary Information 3

Electric field distribution in the sample.

The electric field at the sample and around was calculated by using the finite-elements solver COMSOL Multiphysics® Modeling Software package 4.4, at the device dimensions: a) substrate thickness 0.5 mm, b) lateral dimensions 5 x 5 mm^2, c) contact width 0.5 mm; d) contact length 5 mm that were placed on the crystal surface, opposite to LSMO face, along opposite edges of the substrate.

The calculated electric field (modulus) distribution is shown in Fig. S3. The color barcode indicates the strength of the electric field (V/m) at any point.

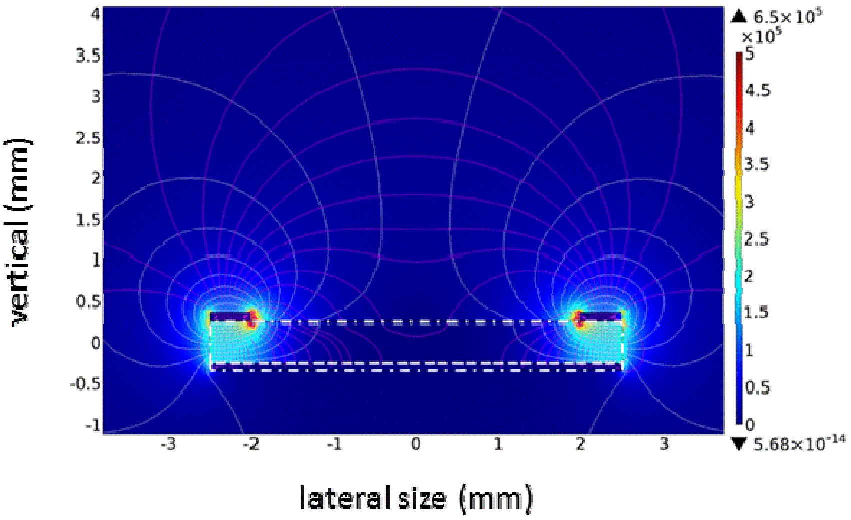

Figure S3. Electric field distribution in the device. Field units in the code bar (right vertical scale) are in V/m. Dimensions are in mm.

Supplementary Information 4

Temperature dependence of the magnetic moment.

In Figure S4 we show the m(T) data for the 34 nm LSMO sample recorded upon warming under H_{meas} = 65 Oe, after an E-FC (V = +210 V) process; at some fixed temperatures during the warming process, the E-field was varied as indicated. For comparison we also include *m*(T) data collected upon warming under V = 0 and H_{meas} = 65 Oe after a E-ZFC process. It can be observed that at the lowest temperatures, the magnetic moment recorded under E ≠ 0 is smaller than the corresponding value recorded under E = 0 This is in agreement with data in Figure 2b (main text). Next, we observe that at 22 K, when the E-field is zeroed, there is a rapid increase of magnetic moment, approaching - but still smaller- the *m*(T, E = 0) data (open symbols). This observation nicely illustrates that E-field steps, irrespectively of its sign, induce changes in the STO affecting the magnetic state of the LSMO, which gradually approaches its equilibrium state.

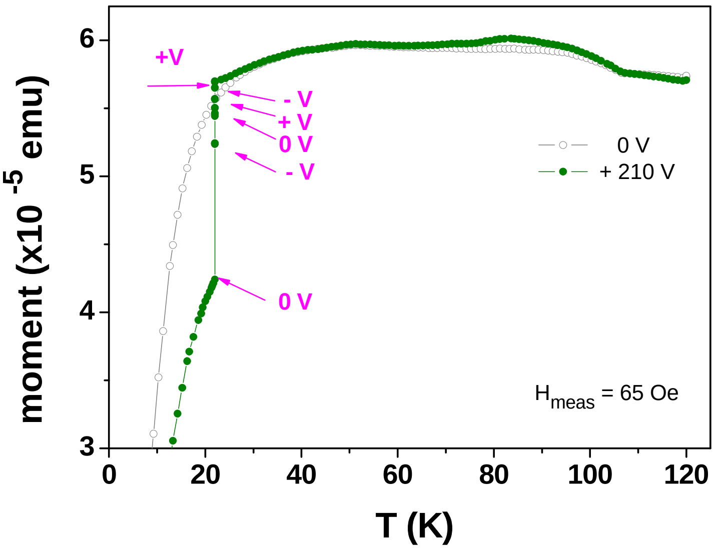

Figure S4|Electric field effects on the magnetic moment at fixed temperature and magnetic field on the LSMO film, on heating. Temperature dependence of the magnetic moment of the 34 nm thick, LSMO film under H_{meas} = 65 Oe. Prior each measurement the sample was cooled down from T >> T_{CT} to 5 K, in magnetic field of -65 Oe, either without E-field applied (open symbols) or under electric field (210 V, solid symbols) and measurements were done upon warming. On sample cooled under E-field (solid symbols), arrows in the warming cycle indicate the V steps performed. First V changed was performed, at 22K, where V was zeroed. Subsequent V changes are indicated.
